# Brain-Derived Neurotrophic Factor Gene Val66Met Polymorphism Modulates Reversible Cerebral Vasoconstriction Syndromes

**DOI:** 10.1371/journal.pone.0018024

**Published:** 2011-03-18

**Authors:** Shih-Pin Chen, Jong-Ling Fuh, Shuu-Jiun Wang, Shih-Jen Tsai, Chen-Jee Hong, Albert C. Yang

**Affiliations:** 1 Department of Neurology, Neurological Institute, Taipei Veterans General Hospital, Taipei, Taiwan; 2 Faculty of Medicine, National Yang-Ming University School of Medicine, Taipei, Taiwan; 3 Department of Psychiatry, Taipei Veterans General Hospital, Taipei, Taiwan; 4 Institute of Clinical Medicine, National Yang-Ming University School of Medicine, Taipei, Taiwan; Istituto Dermopatico dell'Immacolata, Italy

## Abstract

**Background:**

Reversible cerebral vasoconstriction syndrome (RCVS) could be complicated by cerebral ischemic events. Hypothetical mechanisms of RCVS involve endothelial dysfunction and sympathetic overactivity, both of which were reported to be related to brain-derived neurotrophic factor (BDNF). The study investigated the association between functional BDNF Val66Met polymorphism and RCVS.

**Methods:**

Patients with RCVS and controls were prospectively recruited and genotyped for the BDNF Val66Met polymorphism. Magnetic resonance angiography (MRA) and transcranial color-coded Doppler sonography were employed to evaluate cerebral vasoconstriction. Genotyping results, clinical parameters, vasoconstriction scores, mean flow velocities of the middle cerebral artery (V_MCA_), and Lindegaard indices were analyzed. Split-sample approach was employed to internally validate the data.

**Principal Findings:**

Ninety Taiwanese patients with RCVS and 180 age- and gender-matched normal controls of the same ethnicity completed the study. The genotype frequencies did not differ between patients and controls. Compared to patients with Met/Met homozygosity, patients with Val allele had higher mean vasoconstriction scores of all arterial segments (1.60±0.72 vs. 0.87±0.39, p<0.001), V_MCA_ values (116.7±36.2 vs. 82.7±17.9 cm/s, p<0.001), and LI (2.41±0.91 vs. 1.89±0.41, p = 0.001). None of the Met/Met homozygotes, but 38.9% of the Val carriers, had V_MCA_ values of >120 cm/s (p<0.001). Split-sample validation by randomization, age, entry time or residence of patients demonstrated concordant findings.

**Conclusions:**

Our findings link BDNF Val66Met polymorphism with the severity of RCVS for the first time and implicate possible pathogenic mechanisms for vasoconstriction in RCVS.

## Introduction

Reversible cerebral vasoconstriction syndrome (RCVS) is characterized by recurrent severe headaches, mostly thunderclap headaches, and reversible cerebral vasoconstriction [1]–[3]. Cerebral vasoconstriction in RCVS is pervasive, outlasts headache resolution [4], and is the most important component of RCVS. Severe vasoconstrictions, especially in the M1 segment of the middle cerebral artery (MCA) and P2 segment of the posterior cerebral artery (PCA), are associated with higher risks of posterior reversible encephalopathy syndrome (PRES) and ischemic stroke [4], [5]. In addition, a substantial proportion of patients are complicated by hemorrhagic complications such as intracerebral hemorrhage (ICH) or cortical subarachnoid hemorrhage [2], [6]. The pathogenesis of RCVS remains enigmatic, although sympathetic over-activity and endothelial dysfunction are proposed to play important roles. The homogeneity of patient demographic and clinical characteristics suggests a common diathesis; however, the possibility of a genetic predisposition for this disease has never been explored.

Brain-derived neurotrophic factor (BDNF) is important in neuronal survival, neurogenesis, and synaptic plasticity. Like other neurotrophins, BDNF utilizes a dual receptor system to modulate diverse and sometimes opposing biological actions that consists of a specific high affinity receptor [Neurotrophic tyrosine kinase, receptor, type 2 (NTKR2), also known as TrkB (a member of the Trk family of tyrosine kinase receptors)] and a common low affinity receptor [p75 neurotrophin receptor (p75^NTR^)][7]–[9]. In addition to its neurotrophic effects, BDNF also has complex effects on vascular function, promoting angiogenesis through the TrkB receptor expressed on endothelial cells [10] and having possible dual effects on vascular smooth muscle cells through the p75^NTR^ receptor [11]. Besides, BDNF has complex interactions with sympathetic neurons[12], [13] and has been implicated in disorders of vascular tone dysregulation [14].

The human BDNF gene has been mapped to chromosome 11. A common single nucleotide polymorphism (SNP) (G196A or Val66Met, dbSNP: rs6265) in the BDNF gene leading to a valine (Val) to methionine (Met) substitution in codon 66 of the prodomain, has been shown to impact intracellular trafficking and activity-dependent secretion of BDNF [15]. This functional polymorphism was recently found to be associated with unstable angina, supporting a genetic basis for the BDNF-vascular interaction [16]. In this study, we hypothesized that the BDNF Val66Met polymorphism might have modulatory effects on RCVS, especially vasoconstriction.

## Methods

### Participants

Consecutive patients with RCVS were recruited from the headache clinic at Taipei Veterans General Hospital (TVGH) between 2005 and 2010. TVGH is a 2,909-bed national medical center that serves both veterans and non-veteran citizens. This hospital is located in Taipei City, which is both the capital and a major urban center in Taiwan, and had a population of approximately 2,610,000 in 2009. The headache clinic of TVGH has been operating since 1997. The diagnosis of RCVS was based on the criteria of its eponymous syndrome “benign (or reversible) angiography of the central nervous system” proposed by the International Classification of Headache Disorders, second edition (ICHD-2) (Code 6.7.3), except for the duration criterion D [17]. The duration criterion was not followed because some patients took >2 months to recover [3]. Controls were recruited from neurologic and psychiatric clinics as well as from the medical and paramedical staffs of TVGH for comparison.

### Neuroimaging studies

Protocols of magnetic resonance (MR) studies and transcranial color-coded sonography (TCCS) have been detailed elsewhere [4], [5]. Briefly, magnetic resonance angiography (MRA) was obtained using a 3D time-of-flight (TOF) MR technique with multi-slab reconstruction and maximum intensity projection (MIP) postprocessing to assess the extent and reversibility of vasoconstriction. The basilar artery (BA) and the first and second segments of the anterior cerebral artery (ACA, A1 and A2), MCA (M1 and M2), and PCA (P1 and P2) were evaluated. Vasoconstriction severity was graded on a five-point scale, 0 (<10%), 1 (10-<25%), 2 (25-<50%), 3 (50-<75%), and 4 (≥ 75%), and designated as the “vasoconstriction score” of each arterial segment. Only patients with multifocal cerebral vasoconstrictions on the initial MRA and demonstration of the reversibility of vasoconstrictions on a follow-up MRA were eligible. The mean flow velocity of the MCA (V_MCA_) and the Lindegaard index (LI), which was calculated by dividing the V_MCA_ by the mean flow velocity of the ipsilateral distal extracranial internal carotid artery (ICA), were obtained to evaluate the hemodynamic derangement caused by vasoconstriction.

### BDNF polymorphism

Genomic DNA was extracted from EDTA-containing venous blood samples for BDNF Val66Met polymorphism genotyping [18]. The DNA fragments of interest were amplified using polymerase chain reaction (PCR) with the primers 5′-ACTCTGGAGAGCGTGAAT-3′ and 5′-ATACTGTCACACACGCTC-3′. The PCR was performed in a total volume of 10 µl containing 50 ng of template DNA, 50 mM KCl, 10 mM Tris–HCl (pH 8.3), 1.5 mM MgCl_2_, 200 µM of dNTP, 10 pmol of each oligonucleotide, and 0.25 U of *Taq* DNA polymerase. Amplification conditions consisted of an initial 4-min denaturation step at 94°C, 32 cycles of 30 s at 94°C, 30 s at 58°C, and 30 s at 72°C, followed by a final extension of 10 min at 74°C. The Val66Met polymorphism was differentiated with the *Nla*III restriction enzyme. Partial digestion was minimized by an internal restriction site and a control sample of digestible homozygous Val/Val. The genotyping was processed blinded to clinical data.

### Ethics

The study protocol was approved by the TVGH Institutional Review Board. All participants provided written informed consent before entering the study. All clinical investigation was conducted according to the principles expressed in the Declaration of Helsinki. The corresponding author had full access to all the data in the study and had final responsibility for the decision to submit for publication.

### Statistics

In this study, descriptive statistics are presented as the median, mean ± SD, or percentages. Means were compared using the unpaired *t*-tests, and proportions were compared using the Chi-square or Fisher's exact test as appropriate. Genotypes and allele frequency of patients were analyzed and compared with those found in controls. The severity of vasoconstriction (mean vasoconstriction scores, V_MCA_, and LI) [4], [5] was compared between different genotypes in patients with RCVS by analysis of variance (ANOVA) test and post-hoc analysis with Scheffe test. To minimize the risk of false-positive findings, we used a split-sample approach to internally validate the data [19]. The dataset of patients with RCVS was split into two approximately equal subsets by 1∶1 randomization or parameters including median age, median entry time and residence (Taipei City or outside Taipei City) of the patients. All calculated *p*-values were two-tailed, and statistical significance was defined as a *p*-value of <0.01 to adjust type I error resulted from multiple comparisons.

## Results

### Participants and their characteristics

We recruited 90 Taiwanese RCVS patients and 180 age-and sex- matched controls (mean age: 48.1±10.4 vs. 48.2±17.2 year, p = 0.94; Gender ratio: M/F 11/79 vs. 22/158, p = 1.00) with the same ethnicity. The median age of patients was 51. Seventeen patients (18.9%) had a history of hypertension and 39 (43.3%) had blood pressure surges during headache attacks. Five patients (5.6%) had type 2 diabetes mellitus, 18 (20.0%) had migraine, and none had coronary artery disease. Forty (50.6%) women were postmenopausal, and eleven (13.9%) were under hormone therapy at disease onset. Four patients had some possibly associated conditions, including the use of selective serotonin reuptake inhibitors, ingestion of pseudoephedrine, left distal vertebral artery dissection, or microangiopathic hemolytic anemia. Patients had an average of 6.6±6.0 (range 2–30) thunderclap headache attacks in a mean period of 16.8±8.4 d (range 2–42 d). Triggers were identifiable in 71 (78.8%) patients. Five patients (5.6%) developed PRES and five (5.6%) developed ischemic stroke; two had both PRES and ischemic stroke. One patient with both PRES and ischemic stroke was also complicated with ICH.

### Genotypes and allele frequencies between patients and controls

Comparison of the genotypes and allelic frequencies of the BDNF Val66Met polymorphism between patients and controls is presented in Table 1. The distribution of the BDNF Val66Met genotypes was in Hardy–Weinberg equilibrium in patients and controls. Neither genotypes nor allele frequencies were different between patient and control groups.

**Table 1:** Comparison of genotypes and allelic frequencies for the brain-derived neurotrophic factor Val66Met polymorphism between patients with reversible cerebral vasoconstriction syndrome and controls.

Group	Genotypes, n (%)			P	Allele frequency, %		P
	Val/Val	Val/Met	Met/Met		Val	Met	
Patients (n = 90)	19 (21.1)	45 (50.0)	26 (28.9)	0.87	46.1	53.9	1.00
Controls (n = 180)	35 (19.4)	96 (53.3)	49 (27.2)		46.1	53.9	

### Genotyping results versus clinical variables in patients with RCVS

The comparisons of vasoconstriction scores, V_MCA_ and LI between patients with different genotypes are listed in table 2. Patients with Val/Val or Val/Met genotypes had more severe vasoconstrictions than Met/Met carriers, but the severity did not differ between those with Val/Val and Val/Met genotype (Table 2). Post-hoc analysis using the dominant model of inheritance for Val allele disclosed that Val carriers had significantly higher mean vasoconstriction scores of M1, M2, A1, and P2, higher V_MCA_ values, and LI than Met/Met homozygotes (p<0.001 for M1, A1 and V_MCA_; p = 0.001 for M2, P2 and LI). In addition, we found that none of the Met/Met homozygotes had a V_MCA_ value >120 cm/s, compared to 38.9% of Val carriers (*p* <0.001). Clinical features and complication risk were not associated with the polymorphism (Table 2).

**Table 2:** Comparisons of demographics, past medical history, clinical manifestation and vasoconstriction severity of reversible cerebral vasoconstriction syndromes in association with brain-derived neurotrophic factor Val66Met polymorphisms.

	Val/Val(n = 19)	Val/Met(n = 45)	Met/Met(n = 26)	P†
Age (y), mean ± SD	48.9±11.0	48.0±9.5	47.7±11.7	0.924
Gender, M/F	4/15	3/42	4/22	0.238
Hypertension, n (%)	2 (10.5)	8 (17.8)	7 (26.9)	0.376
Type 2 DM, n (%)	1 (5.3)	2 (4.4)	1(7.7)	0.850
Migraine, n (%)	3 (15.8)	10 (22.2)	5 (19.2)	0.840
BP surge, n (%)	7 (36.8)	22 (48.9)	10 (38.4)	0.574
Total TCH attacks, mean ± SD	9.9±10.9	6.2±4.2	5.2±3.4	0.097
TCH duration (d), mean ± SD	17.5±10.3	16.6±11.1	16.7±13.4	0.970
Mean vasoconstriction score, mean ± SD				
M1	1.24±1.02*	1.78±1.03*	0.58±0.52	<0.001
M2	1.53±1.42	2.17±1.04**	1.12±0.90	0.001
A1	2.00±1.13**	1.95±0.97**	0.90±0.60	<0.001
A2	1.34±0.97	1.47±0.91	0.96±0.69	0.064
P1	1.39±0.95	1.37±1.04	0.83±0.78	0.057
P2	2.03±1.09*	1.92±0.92*	1.19±0.98	0.005
BA	0.63±0.90	0.70±0.93	0.31±0.62	0.160
All segments	1.43±0.73*	1.70±0.70*	0.87±0.39	<0.001
V_MCA_	111.1±33.3*	113.4±38.0**	82.7±17.9	0.003
LI	2.32±0.79	2.45±0.97	1.89±0.41	0.050
PRES, n (%)	0 (0)	4 (9)	1 (4)	0.338
Ischemic stroke, n (%)	0 (0)	3 (7)	2 (8)	0.493

BA: basilar artery, BP: blood pressure, DM: diabetes mellitus, LI: Lindegaard index, PRES: posterior reversible encephalopathy syndrome, TCH: thunderclap headache, V_MCA_: mean flow velocity of the middle cerebral artery.

† Derived by analysis of variance (ANOVA) test.

*p<0.05 & **p<0.01 in comparison with Met/Met homozygotes using post-hoc Scheffe test.

### Split-sample internal validation

When the dataset was randomly split in half, we found that Val carriers (Val/Val or Val/Met genotypes) had higher vasoconstriction scores than Met/Met homozygotes in both subgroups (Table S1). The demographics and genotyping frequencies did not differ between these two subgroups (complete data not shown). When we divided the sample by median age, median entry time and residence as defined, we discovered concordant findings among these divided subsets (Tables S1, S2, S3, S4).

## Discussion

Although the disease profile of RCVS has been delineated, the underlying mechanisms are largely unknown. Our results showed that the Val allele in codon 66 of the BDNF gene was associated with more severe vasoconstriction in patients with RCVS, or on the contrary, the presence of Met homozygosity had a protective role. This is the first study to unravel possible genetic substrate of this highly homogeneous clinical-radiological syndrome. Though there is still a long way to go, this novel finding provides a key to unlock the pathogenetic networks of this potentially fatal disease.

Because RCVS is not a common disease, accounting for less than 1% of patients in our headache clinic [3], the case number of this five-year study has been the largest ever since. To validate our findings by repeating the same results in another sample with a similar size is difficult. Thus, our study employed a split-sample approach for internal validation. Of note, splitting one well-powered study frequently results in two less conclusive ones [20]. However, even with this trade-off, we obtained similar findings in different split subsets. Therefore, the genetic association of BDNF Val66Met is unlikely a false-positive finding.

A previous study investigating the relationship between unstable angina and the BDNF Val66Met polymorphism similarly identified a protective effect of the Met/Met genotype [16]. Though the pathogeneses of RCVS and unstable angina might differ, the common findings of both studies suggest the benefit of further BDNF studies in vascular disorders. Because the allelic frequency was similar between our patients and controls, BDNF Val66Met polymorphism was not likely to be associated with the genetic susceptibility to RCVS. It should be noted that if the BDNF-gene Val66Met polymorphism is an uncommon disease locus or one with a small effect, the failure to demonstrate a significant association may reflect false negative results due to a small sample size. It is also possible that another BDNF genetic variant is involved in susceptibility to RCVS, and that the linkage disequilibrium between this genetic variant and the Val66Met polymorphism did not occur in our study population.

The allele frequencies of the BDNF Val66Met polymorphism differ among different ethnic populations. The frequency of Met66 allele varies from 0 to 72% among 58 global populations; it is considerably low in African or Caucasian populations but higher in Asian descents [21]. This might implicate that patients in western countries are more vulnerable to severe vasoconstrictions, and therefore, more complications than Asian patients. However, whether the results of our study could be generalizable to other ethnic populations deserve further investigation. Since all cases and controls in this study were of the same ethnicity and our observed allele frequencies are consistent with those observed for Han Chinese, population stratification is unlikely to play a major confounding role in our study. Nonetheless, the difference in polymorphism frequency could be a major issue for subsequent replication studies with different ethnic backgrounds.

The findings of this study raise the significance of potential biologic actions of BDNF polymorphism on cerebral vasoconstriction. If the findings could be validated with consistent and independent replication, we might be able to identify patients at a higher risk of severe vasoconstrictions in the era of personalized medicine. The mechanisms underlying our findings are not known. The followings are some possible explanations. First, earlier study demonstrated that depolarization-induced secretion was reduced in 66Met BDNF-transfected neurons compared with 66Val BDNF analogs [15]. Thus subjects carrying Val allele may have higher intracranial BDNF activity than Met/Met homozygotes. Second, it has been reported that under circumstances of sympathetic overactivity, BDNF could lead to perivascular inflammation and thus cause marked vasoconstriction [14]. BDNF has been shown to dramatically upregulate neuropeptide Y [22], a vasoconstrictive sympathetic co-transmitter [23]. Since sympathetic overactivity may play an important role in the pathogenesis of RCVS [24], it is plausible that subjects with higher intracranial BDNF activity, i.e., the Val carriers, could have more severe vasoconstriction. Third, BDNF-overexpression was found to modulate vascular tone of small pulmonary arteries through interactions with neurokinin A [25]. Because receptor subtypes of neurokinin A differ between cerebral and pulmonary arteries, whether the study results could be extrapolated to RCVS requires further studies.

### Limitations

Our study had several limitations. First, the case number in this study was not large for a genetic polymorphism study and might have led to false negative results of clinical correlates, such as thunderclap attacks or total duration. However, the positive results with large effect sizes alleviated potential type II errors caused by small sample size. Second, all the patients with V_MCA_ >120 cm/s were Val carriers. Based on our previous study [5], the risks of PRES or ischemic stroke in these patients should be higher. However, because of limited case number with complications in the present study cohort, we failed to observe this expected finding. One reason for lower percentage of patients with complications could be our improving ability of early recognition and treatment for RCVS. Third, we did not check plasma BDNF levels in patients, and were unable to ensure whether the modulatory effect of this polymorphism came from an altered BDNF level and/or its downstream pathways. Nonetheless, recent studies demonstrated that plasma concentration of BDNF was not associated with the Val66Met variant [26] and there was no significant correlation between serum and regional brain BDNF levels [27]. Furthermore, the possibility that the actual causal SNP was not the Val66Met polymorphism per se but located at a highly linked locus could not be completely excluded. Large-scaled studies with haplotype analysis or direct sequencing might be required to answer the question in the future.

## Supporting Information

Table S1 **Split sample by 1∶1 randomization (part 1 and 2).** Comparison of vasoconstriction severity between Val carriers and Met homozygotes.
(DOC)

Table S2 **Split sample by median age (part 1: age≦51; part 2: age >51).** Comparison of vasoconstriction severity between Val carriers and Met homozygotes.
(DOC)

Table S3 **Split sample by median entry time (part 1: before 2009; part 2: since 2009).** Comparison of vasoconstriction severity between Val carriers and Met homozygotes.
(DOC)

Table S4 **Split sample by patient residence (part 1: Taipei City; part 2: outside Taipei City).** Comparison of vasoconstriction severity between Val carriers and Met homozygotes.
(DOC)

## References

[1] Calabrese LH, Dodick DW, Schwedt TJ, Singhal AB (2007). Narrative review: reversible cerebral vasoconstriction syndromes.. Ann Intern Med.

[2] Ducros A, Boukobza M, Porcher R, Sarov M, Valade D (2007). The clinical and radiological spectrum of reversible cerebral vasoconstriction syndrome. A prospective series of 67 patients.. Brain.

[3] Chen SP, Fuh JL, Lirng JF, Chang FC, Wang SJ (2006). Recurrent primary thunderclap headache and benign CNS angiopathy: spectra of the same disorder?. Neurology.

[4] Chen SP, Fuh JL, Wang SJ, Chang FC, Lirng JF (2010). Magnetic resonance angiography in reversible cerebral vasoconstriction syndromes.. Ann Neurol.

[5] Chen SP, Fuh JL, Chang FC, Lirng JF, Shia BC (2008). Transcranial color doppler study for reversible cerebral vasoconstriction syndromes.. Ann Neurol.

[6] Kumar S, Goddeau RP, Selim MH, Thomas A, Schlaug G (2010). Atraumatic convexal subarachnoid hemorrhage: clinical presentation, imaging patterns, and etiologies.. Neurology.

[7] Pezet S, Malcangio M (2004). Brain-derived neurotrophic factor as a drug target for CNS disorders.. Expert Opin Ther Targets.

[8] Arévalo JC, Wu SH (2006). Neurotrophin signaling: many exciting surprises! Cell Mol Life Sci.

[9] Huang EJ, Reichardt LF (2001). Neurotrophins: roles in neuronal development and function.. Annu Rev Neurosci.

[10] Kermani P, Hempstead B (2007). Brain-derived neurotrophic factor: a newly described mediator of angiogenesis.. Trends Cardiovasc Med.

[11] Wang S, Bray P, McCaffrey T, March K, Hempstead BL (2000). p75(NTR) mediates neurotrophin-induced apoptosis of vascular smooth muscle cells.. Am J Pathol.

[12] Yang B, Slonimsky JD, Birren SJ (2002). A rapid switch in sympathetic neurotransmitter release properties mediated by the p75 receptor.. Nat Neurosci.

[13] Slonimsky JD, Yang B, Hinterneder JM, Nokes EB, Birren SJ (2003). BDNF and CNTF regulate cholinergic properties of sympathetic neurons through independent mechanisms.. Mol Cell Neurosci.

[14] Kasselman LJ, Sideris A, Bruno C, Perez WR, Cai N (2006). BDNF: a missing link between sympathetic dysfunction and inflammatory disease?. J Neuroimmunol.

[15] Egan MF, Kojima M, Callicott JH, Goldberg TE, Kolachana BS (2003). The BDNF val66met polymorphism affects activity-dependent secretion of BDNF and human memory and hippocampal function.. Cell.

[16] Jiang H, Wang R, Liu Y, Zhang Y, Chen ZY (2009). BDNF Val66Met polymorphism is associated with unstable angina.. Clin Chim Acta.

[17] Headache Classification Subcommittee of the International Headache Society (2004). The International Classification of Headache Disorders: 2nd edition.. Cephalalgia.

[18] Hong CJ, Yu YW, Lin CH, Tsai SJ (2003). An association study of a brain-derived neurotrophic factor Val66Met polymorphism and clozapine response of schizophrenic patients.. Neurosci Lett.

[19] Picard RR, Berk KN (1990). Data splitting.. Am Stat.

[20] Chanock SJ, Manolio T, Boehnke M, Boerwinkle E, Hunter DJ (2007). Replicating genotype-phenotype associations.. Nature.

[21] Petryshen TL, Sabeti PC, Aldinger KA, Fry B, Fan JB (2010). Population genetic study of the brain-derived neurotrophic factor (BDNF) gene.. Mol Psychiatry.

[22] Croll SD, Wiegand SJ, Anderson KD, Lindsay RM, Nawa H (1994). Regulation of neuropeptides in adult rat forebrain by the neurotrophins BDNF and NGF.. Eur J Neurosci.

[23] Zukowska Z, Pons J, Lee EW, Li L (2003). Neuropeptide Y: a new mediator linking sympathetic nerves, blood vessels and immune system?. Can J Physiol Pharmacol.

[24] Dodick DW (2002). Thunderclap headache.. J Neurol Neurosurg Psychiatry.

[25] Springer J, Wagner S, Subramamiam A, McGregor GP, Groneberg DA (2004). BDNF-overexpression regulates the reactivity of small pulmonary arteries to neurokinin A.. Regul Pept.

[26] Terracciano A, Martin B, Ansari D, Tanaka T, Ferrucci L (2010). Plasma BDNF concentration, Val66Met genetic variant and depression-related personality traits.. Genes Brain Behav.

[27] Luo KR, Hong CJ, Liou YJ, Hou SJ, Huang YH (2010). Differential regulation of neurotrophin S100B and BDNF in two rat models of depression.. Prog Neuropsychopharmacol Biol Psychiatry.

