# Supplementary material for: Brain-Derived Neurotrophic Factor Gene Val66Met Polymorphism Modulates Reversible Cerebral Vasoconstriction Syndromes
Source: PLoS One. 2011 Mar 18;6(3):e18024. doi: 10.1371/journal.pone.0018024 (PMC3060928; doi:10.1371/journal.pone.0018024)
Supplement: Table S1 — Split sample by 1∶1 randomization (part 1 and 2). Comparison of vasoconstriction severity between Val carriers and Met homozygotes. (DOC) [file pone.0018024.s001.doc]

Table S1. Split sample by 1:1 randomization (part 1 and 2). Comparison of vasoconstriction severity between Val carriers and Met homozygotes.

|  | Part 1 | | | p | Part 2 | | | p |
| --- | --- | --- | --- | --- | --- | --- | --- | --- |
|  | Val carriers  (n=30) | Met/Met  homozygotes  (n=15) | |  | Val carriers  (n=34) | Met/Met  homozygotes  (n=11) | |  |
| Mean vasoconstriction score, mean ± SD | | | | | | | | |
| M1 | 1.32 ± 0.93 | | 0.50 ± 0.53 | 0.001 | 1.88 ± 1.09 | | 0.68 ± 0.51 | <0.001 |
| M2 | 1.77 ± 1.19 | | 1.13 ± 0.79 | 0.040 | 2.16 ± 1.25 | | 1.09 ± 1.07 | 0.010 |
| A1 | 1.90 ± 1.02 | | 0.90 ± 0.66 | 0.001 | 2.03 ± 1.01 | | 0.90 ± 0.52 | <0.001 |
| A2 | 1.35 ± 0.88 | | 0.73 ± 0.68 | 0.022 | 1.50 ± 0.96 | | 1.27 ± 0.61 | 0.466 |
| P1 | 1.22 ± 0.98 | | 0.78 ± 0.66 | 0.128 | 1.53 ± 1.02 | | 0.90 ± 0.97 | 0.091 |
| P2 | 2.05 ± 1.01 | | 1.23 ± 1.13 | 0.018 | 1.87 ± 0.92 | | 1.14 ± 0.78 | 0.023 |
| BA | 0.76 ± 0.99 | | 0.33 ± 0.72 | 0.148 | 0.62 ± 0.85 | | 0.27 ± 0.47 | 0.099 |
| All segments | 1.50 ± 0.70 | | 0.85 ± 0.39 | 0.001 | 1.70 ± 0.73 | | 0.90 ± 0.41 | <0.001 |
| V_MCA_ | 111.8 ± 36.7 | | 88.8 ± 19.4 | 0.028 | 113.4 ± 36.4 | | 77.7 ± 15.6 | <0.001 |
| LI | 2.57 ± 1.09 | | 1.91 ± 0.50 | 0.091 | 2.28 ± 0.73 | | 1.88 ± 0.34 | 0.022 |

BA: basilar artery, LI: Lindegaard index, V_MCA_: mean flow velocity of the middle cerebral artery.
