# Supplementary material for: Brain-Derived Neurotrophic Factor Gene Val66Met Polymorphism Modulates Reversible Cerebral Vasoconstriction Syndromes
Source: PLoS One. 2011 Mar 18;6(3):e18024. doi: 10.1371/journal.pone.0018024 (PMC3060928; doi:10.1371/journal.pone.0018024)
Supplement: Table S2 — Split sample by median age (part 1: age≦51; part 2: age >51). Comparison of vasoconstriction severity between Val carriers and Met homozygotes. (DOC) [file pone.0018024.s002.doc]

Table S2. Split sample by median age (part 1: age≦51; part 2: age > 51). Comparison of vasoconstriction severity between Val carriers and Met homozygotes.

|  | Part 1 | | | p | Part 2 | | | p |
| --- | --- | --- | --- | --- | --- | --- | --- | --- |
|  | Val carriers  (n=32) | Met/Met  homozygotes  (n=13) | |  | Val carriers  (n=32) | Met/Met  homozygotes  (n=13) | |  |
| Mean vasoconstriction score, mean ± SD | | | | | | | | |
| M1 | 1.81 ± 1.07 | | 0.77 ± 0.56 | <0.001 | 1.42 ± 1.01 | | 0.38 ± 0.42 | <0.001 |
| M2 | 1.98 ± 1.20 | | 1.35 ± 0.94 | 0.095 | 1.97 ± 1.20 | | 0.88 ± 0.82 | 0.005 |
| A1 | 1.84 ± 1.14 | | 1.00 ± 0.64 | 0.004 | 2.10 ± 0.86 | | 0.81 ± 0.56 | <0.001 |
| A2 | 1.69 ± 0.90 | | 1.15 ± 0.80 | 0.071 | 1.17 ± 0.88 | | 0.77 ± 0.53 | 0.066 |
| P1 | 1.47 ± 1.09 | | 1.10 ± 0.95 | 0.310 | 1.28 ± 0.92 | | 0.58 ± 0.49 | 0.002 |
| P2 | 2.02 ± 1.06 | | 1.62 ± 0.87 | 0.235 | 1.89 ± 0.87 | | 0.77 ± 0.93 | 0.001 |
| BA | 0.84 ± 1.07 | | 0.31 ± 0.63 | 0.048 | 0.53 ± 0.72 | | 0.31 ± 0.63 | 0.333 |
| All segments | 1.71 ± 0.80 | | 1.04 ± 0.38 | 0.002 | 1.50 ± 0.62 | | 0.71 ± 0.35 | <0.001 |
| V_MCA_ | 120.3 ± 41.0 | | 85.3 ± 14.7 | <0.001 | 103.0 ± 27.1 | | 78.8 ± 22.3 | 0.029 |
| LI | 2.57 ± 1.07 | | 1.95 ± 0.39 | 0.010 | 2.21 ± 0.61 | | 1.80 ± 0.44 | 0.090 |

BA: basilar artery, LI: Lindegaard index, V_MCA_: mean flow velocity of the middle cerebral artery.
