# Supplementary material for: Brain-Derived Neurotrophic Factor Gene Val66Met Polymorphism Modulates Reversible Cerebral Vasoconstriction Syndromes
Source: PLoS One. 2011 Mar 18;6(3):e18024. doi: 10.1371/journal.pone.0018024 (PMC3060928; doi:10.1371/journal.pone.0018024)
Supplement: Table S3 — Split sample by median entry time (part 1: before 2009; part 2: since 2009). Comparison of vasoconstriction severity between Val carriers and Met homozygotes. (DOC) [file pone.0018024.s003.doc]

Table S3. Split sample by median entry time (part 1: before 2009; part 2: since 2009). Comparison of vasoconstriction severity between Val carriers and Met homozygotes.

|  | Part 1 | | | p | Part 2 | | | p |
| --- | --- | --- | --- | --- | --- | --- | --- | --- |
|  | Val carriers  (n=32) | Met/Met  homozygotes  (n=13) | |  | Val carriers  (n=32) | Met/Met  homozygotes  (n=13) | |  |
| Mean vasoconstriction score, mean ± SD | | | | | | | | |
| M1 | 1.38 ± 1.00 | | 0.58 ± 0.53 | 0.001 | 1.86 ± 1.06 | | 0.58 ± 0.53 | <0.001 |
| M2 | 1.88 ± 1.34 | | 1.19 ± 0.79 | 0.027 | 2.08 ± 1.25 | | 1.04 ± 1.03 | 0.011 |
| A1 | 1.94 ± 0.97 | | 0.96 ± 0.66 | 0.002 | 2.00 ± 1.06 | | 0.83 ± 0.54 | 0.001 |
| A2 | 1.27 ± 0.96 | | 0.88 ± 0.71 | 0.203 | 1.59 ± 0.87 | | 1.04 ± 0.69 | 0.046 |
| P1 | 1.13 ± 1.03 | | 0.64 ± 0.59 | 0.135 | 1.66 ± 0.91 | | 1.00 ± 0.91 | 0.037 |
| P2 | 1.98 ± 0.93 | | 1.50 ± 1.11 | 0.142 | 1.92 ± 1.01 | | 0.88 ± 0.74 | 0.002 |
| BA | 0.66 ± 0.79 | | 0.46 ± 0.78 | 0.454 | 0.71 ± 1.04 | | 0.15 ± 0.38 | 0.013 |
| All segments | 1.42 ± 0.73 | | 0.92 ± 0.38 | 0.012 | 1.78 ± 0.68 | | 0.81 ± 0.42 | <0.001 |
| V_MCA_ | 115.2 ± 31.4 | | 82.6 ± 18.6 | 0.006 | 109.7 ± 41.5 | | 82.8 ± 18.1 | 0.011 |
| LI | 2.30 ± 0.71 | | 1.79 ± 0.25 | 0.002 | 2.53 ± 1.10 | | 1.98 ± 0.49 | 0.044 |

BA: basilar artery, LI: Lindegaard index, V_MCA_: mean flow velocity of the middle cerebral artery.
