# Supplementary material for: Brain-Derived Neurotrophic Factor Gene Val66Met Polymorphism Modulates Reversible Cerebral Vasoconstriction Syndromes
Source: PLoS One. 2011 Mar 18;6(3):e18024. doi: 10.1371/journal.pone.0018024 (PMC3060928; doi:10.1371/journal.pone.0018024)
Supplement: Table S4 — Split sample by patient residence (part 1: Taipei City; part 2: outside Taipei City). Comparison of vasoconstriction severity between Val carriers and Met homozygotes. (DOC) [file pone.0018024.s004.doc]

Table S4. Split sample by patient residence (part 1: Taipei City; part 2: outside Taipei City). Comparison of vasoconstriction severity between Val carriers and Met homozygotes.

|  | Part 1 | | | p | Part 2 | | | p |
| --- | --- | --- | --- | --- | --- | --- | --- | --- |
|  | Val carriers  (n=31) | Met/Met  homozygotes  (n=15) | |  | Val carriers  (n=33) | Met/Met  homozygotes  (n=11) | |  |
| Mean vasoconstriction score, mean ± SD | | | | | | | | |
| M1 | 1.56 ± 0.91 | | 0.43 ± 0.46 | <0.001 | 1.67 ± 1.18 | | 0.77 ± 0.56 | 0.002 |
| M2 | 1.87 ± 1.12 | | 0.97 ± 0.79 | 0.007 | 2.08 ± 1.26 | | 1.32 ± 1.03 | 0.080 |
| A1 | 1.94 ± 1.04 | | 0.93 ± 0.47 | <0.001 | 2.00 ± 1.00 | | 0.86 ± 0.74 | 0.001 |
| A2 | 1.53 ± 0.93 | | 0.80 ± 0.62 | 0.008 | 1.33 ± 0.92 | | 1.18 ± 0.75 | 0.623 |
| P1 | 1.44 ± 1.09 | | 0.63 ± 0.67 | 0.004 | 1.32 ± 0.92 | | 1.12 ± 0.88 | 0.559 |
| P2 | 1.98 ± 0.91 | | 0.60 ± 0.57 | <0.001 | 1.92 ± 1.02 | | 2.00 ± 0.87 | 0.826 |
| BA | 0.68 ± 0.94 | | 0.20 ± 0.56 | 0.038 | 0.69 ± 0.90 | | 0.45 ± 0.69 | 0.437 |
| All segments | 1.62 ± 0.65 | | 0.70 ± 0.27 | <0.001 | 1.58 ± 0.79 | | 1.08 ± 0.43 | 0.022 |
| V_MCA_ | 109.8 ± 30.6 | | 90.2 ± 17.2 | 0.053 | 115.7 ± 41.8 | | 73.6 ± 14.8 | <0.001 |
| LI | 2.32 ± 0.82 | | 2.02 ± 0.41 | 0.263 | 2.51 ± 1.00 | | 1.73 ± 0.36 | 0.002 |

BA: basilar artery, LI: Lindegaard index, V_MCA_: mean flow velocity of the middle cerebral artery.
